# Colorectal Cancer and the Obese Patient: A Call for Guidelines

**DOI:** 10.3390/cancers14215255

**Published:** 2022-10-26

**Authors:** Nikoletta A. Petrou, Henna Rafique, Shahnawaz Rasheed, Paris Tekkis, Christos Kontovounisios

**Affiliations:** 1 Department of General Surgery, The Royal Marsden Hospital, London SW3 6JJ, UK; 2 Department of Surgery and Cancer, Imperial College London, London SW7 2BX, UK; 3 Department of General Surgery, Chelsea and Westminster Hospital, London SW10 9NH, UK

**Keywords:** colorectal cancer, obesity, minimally invasive surgery, surveillance

## Abstract

**Simple Summary:**

Obese patients are known to be at higher risk of developing colorectal cancer. Meanwhile, the rate of obesity continues to rise worldwide. Current guidelines by the National Comprehensive Cancer Network^®^ (NCCN^®^), the European Society of Medical Oncology (ESMO), and the Japanese Society for Cancer of the Colon and Rectum (JSCCR) are not modified to account for the needs of obese patients with colorectal cancer. In this study we aimed to review and compare the existing guidelines and make recommendations specific to this group of patients. We proposed changes in the diagnostic work-up, follow-up and surveillance, perioperative pathways, and management of metastatic disease, with an emphasis on minimally invasive surgical procedures. We concluded that there is need to modify the existing colorectal cancer guidelines to address the needs of obese patients and recommend that a multidisciplinary approach, with involvement of bariatric principles, should be considered.

**Abstract:**

The link between obesity and colorectal cancer has been well established. The worldwide rise in obesity rates in the past 40 years means that we are dealing with increasing numbers of obese patients with colorectal cancer. We aimed to review the existing guidelines and make recommendations specific to this group of patients. Upon comparing the current NCCN Clinical Practice Guidelines in Oncology (NCCN Guidelines ®), the guidelines from the European Society of Medical Oncology (ESMO) and the guidelines of the Japanese Society for Cancer of the Colon and Rectum (JSCCR), we observed that these did not take into consideration the needs of obese patients. We proceeded to make specific recommendations with regards to the diagnostic work-up, surgical pathways, minimally invasive technique, perioperative treatment, post-operative surveillance, and management of metastatic disease in this group of patients. Our review highlights the need for modification of the existing guidelines to account for the needs of this patient cohort. A multidisciplinary approach, including principles used by bariatric surgeons, should be the way forward to reach consensus in the management of this group of patients.

## 1. Introduction

Obesity is a continually rising global phenomenon, with worldwide obesity rates having tripled since 1975. According to the World Health Organisation (WHO), 39% of adults measured as overweight and 13% as obese by 2016 [1]. Obesity is defined as Body Mass Index (BMI) greater than or equal to 30 [2].

Meanwhile, colorectal cancer remains the fourth most common cancer in the UK, with 42,886 new cases diagnosed each year [3]. There is a well-established positive association between obesity and the risk of developing colorectal cancer [4,5,6,7,8], with 11% of colorectal cancer cases being linked directly to being overweight or obese [9].

The use of elective minimally invasive colorectal cancer procedures (MICCP), such as the laparoscopic or robotic approach, is also well-established and increasingly more popular amongst colorectal surgeons in the developed world. The well-described benefits of this approach, (such as reduced length of stay, reduced post-operative pain, lower incidence of surgical site infections, and early mobilisation) are undoubtedly key to an uncomplicated post-surgical recovery of the clinically obese patient [10,11] and are widely preferred by colorectal surgeons when operating on this population. A systematic review and meta-analysis in 2019 examined the safety of open versus laparoscopic colorectal surgery in the obese population. They noted that the laparoscopic approach was overall safe, with no difference in 5-year disease free survival, overall survival, and recurrence rates [12].

In obese patients, there are multiple additional issues that should be taken into consideration when planning their surgical and non-surgical treatment. Obesity has been linked to increased rates of diabetes mellitus, cardiovascular disease, and venous thromboembolism and increased surgical site infections. Understanding the differences in physiology, metabolism, anaesthetic needs, and susceptibility to post-operative complications, as well as the technical challenges associated with MICCP in this group of patients, is therefore of the outmost importance. Surgical lessons can be learned from already-established bariatric surgery pathways and practices and applied to colorectal minimally invasive surgery.

The current NCCN Guidelines^®^, the European Society of Medical Oncology (ESMO) guidelines, and the Japanese Society for Cancer of the Colon and Rectum (JSCCR) guidelines for the management of colon and rectal cancer are not adjusted to specifically account for the obese patient. Therefore, the need for modifications of the current guidelines is imperative to establish a baseline and consensus of how to approach this group of patients. We aimed to review and compare the existing NCCN, ESMO, and JSCCR Guidelines to identify areas where modifications could be made to account for the obese patient.

## 2. Materials and Methods

The Websites of NCCN [13,14], ESMO [15,16,17], and JSCCR [18] were accessed to seek the most up-to-date available guidelines on the topics of colon, rectal, and colorectal cancer.

Data of interest included: year of publication, condition assessed, diagnostic work-up and surgery, pre-operative (neoadjuvant) and postoperative (adjuvant) treatment, post-operative surveillance (follow-up), management of metastatic disease, and current guidelines on minimally invasive surgery. The guidelines were also reviewed for any references to obesity. The guidelines were reviewed by two authors (N.P. and H.R.), who independently identified discrepancies and areas of further research. These were discussed and agreed upon with all the authors. The authors subsequently made recommendations in areas where the current guidelines could be adjusted to account for the obese population.

## 3. Results

### 3.1. Guideline Review

The NCCN Guidelines for colon cancer (2022) and rectal cancer (2022), the JSCCR guidelines for colorectal cancer (2019), and the ESMO guidelines for localised colon cancer (2020), metastatic colorectal cancer (2016), and rectal cancer (2017) were accessed on their respective websites and included in this comparison. Tables 1–9 summarise the findings for each condition. 

### 3.2. Colorectal Polyps with Invasive Cancer

The recommendations are summarised in Table 1 and Table 2. The guidelines recommend that the work-up of colonic and rectal polyps should include tissue diagnosis, colonoscopy, rigid (or flexible) sigmoidoscopy marking of the cancerous polyp site, endoscopic rectal ultrasound (ERUS), and pelvic MRI if applicable. The choice of modality depends on the location (colonic versus rectal) and index of suspicion of the polyp. The guidelines do not include comments on the work-up of the obese patient.

The management of colonic polyps with invasive cancer depends on whether they have been completely removed endoscopically at the time of the colonoscopy and on the morphological features of the polyp. Low-risk polyps are managed by observation, whereas higher-risk polyps would be considered for colectomy with regional lymphadenectomy (NCCN, ESMO). JCSSR recommends polypectomy or snare endoscopic mucosal resection (EMR) for polyps less than 2 cm and endoscopic submucosal dissection (ESD) for polyps 2–5 cm. NCCN recommends transanal local excision for sessile polyps and for those that that have unfavourable histological features or completeness of excision cannot be confirmed. ESMO also recommend transanal local procedures depending on the submucosal (Sm) depth of invasion of the polyp. Guidelines agree that, for higher risk polyps, the approach should be with surgical resection (total mesorectal excision, TME). ESMO recommends chemoradiotherapy or radiotherapy if surgery is contraindicated. The guidelines do not include comments on the obese patient.

### 3.3. Colon and Rectal Cancer (Non-Metastatic)

The recommendations are summarised in Table 1 and Table 2. As part of the diagnostic work-up of colon cancer, NCCN and ESMO recommend pathology review, tumour marker testing (carcinoembryonic antigen, CEA), colonoscopy, and CT chest-abdomen-pelvis. In the case of imaging for rectal cancer, NCCN recommends CT chest and CT or MRI abdomen. NCCN and ESMO also recommend pelvic MRI and NCCN recommends ERUS if MRI is contraindicated, inconclusive, or for superficial lesions. The guidelines agree that CT-PET is not indicated. JCSSR does not formally state recommendations for the work-up of non-metastatic colorectal cancer. The guidelines do not include comments on the obese patient.

The management of non-metastatic colorectal cancer depends on whether the cancer is resectable or unresectable. ESMO recommends local excision for early (Tis/T1 N0) colon cancers. For resectable colon cancers, the standard approach is colectomy with regional lymphadenectomy. NCCN recommends consideration of neoadjuvant chemotherapy or immunotherapy for advanced disease. JSCCR makes recommendations for the extent of the lymphadenectomy depending on cancer staging. NCCN, ESMO, and JSCCR recommend transanal local excision for early rectal cancers (T1 N0). Higher-risk lesions require surgery (TME). Total neoadjuvant therapy, neoadjuvant short-course radiotherapy (SCRT) or chemoradiotherapy (CRT) are recommended for higher-risk lesions (see detailed breakdown in Table 1 and Table 2). The guidelines do not include comments on the obese patient.

### 3.4. Adjuvant Treatment after Curative Resection

Delivery of adjuvant treatment is decided based on post-operative histological staging and the presence of high-risk features (e.g., positive margins, lymphovascular invasion, grade of differentiation). Early low-risk Stage I tumours do not require adjuvant treatment and can be surveyed. The NCCN, ESMO, and JSCCR recommendations on different adjuvant protocols (e.g., chemotherapy, radiotherapy) and their indications according to staging are summarised in Table 3 and Table 4. The guidelines do not include comments on the obese patient.

### 3.5. Postoperative Surveillance

For patients that have completed treatment for colorectal cancer and have entered surveillance, NCCN, ESMO, and JSCCR recommend follow-up with physical examination, monitoring of CEA levels, CT chest-abdomen-pelvis, and colonoscopy. For patients who have entered surveillance post-transanal local excision of rectal cancer, NCCN additionally recommends follow up with proctoscopy with EUS or MRI with contrast. The protocol and time intervals for the surveillance schedule as recommended by each guideline are summarised in Table 5 and Table 6. The guidelines do not include comments on the surveillance of the obese patient.

### 3.6. Metastatic Disease

Synchronous metastatic disease in the liver and lung can be resectable or unresectable. The NCCN, ESMO, and JSCCR recommendations are summarised in Table 7 and Table 8. Surgical resection of lung and liver metastases (that are amenable to surgery) may itself be either synchronous (liver or lung resection at the time of bowel resection surgery) or metachronous (staged). The recommendations for the role of perioperative treatments as well as the role of local ablative techniques are also outlined in Table 7 and Table 8. The guidelines do not include comments on the management of liver and lung metastatic disease in the obese patient.

In patients with peritoneal disease, NCCN recommends palliative surgery (e.g., diverting ostomy, resection, bypass) or stenting in patients with obstructing or imminently obstructing colorectal primary, followed by systemic therapy. NCCN and ESMO recommend that, in appropriate patients, cytoreductive surgery and/or hyperthermic intraperitoneal chemotherapy (HIPEC) can be considered. HIPEC has high morbidity and should be performed in experienced centres with the appropriate set-up. The guidelines do not include comments on the management of peritoneal metastatic disease in the obese patient.

### 3.7. Minimally Invasive Surgery

The recommendations are summarised in Table 9. The NCCN, ESMO, and JSCCR guidelines advise that minimally invasive surgery should be considered based on surgical expertise and skill, tumour location, and staging (e.g., not recommended in locally advanced disease). The ESMO and JSCCR recommendations specifically call attention to patients with previous open surgery (risk of adhesions) and obese patients, as a deterrent when considering a minimally invasive approach but do not specify any absolute contraindications. The recommendations across the three guidelines focus on laparoscopic surgery and do not comment or offer any recommendations on the use of robotic techniques.

## 4. Discussion

In this review of the available guidelines, we observed that overall, the recommendations of the current NCCN, ESMO, and JSCCR Guidelines do not account for the needs of the obese patients in areas such as diagnostic work-up and management of colorectal polyps with invasive cancer, colorectal cancer, adjuvant therapy, surveillance protocols, management of metastatic disease, and minimally invasive surgery. The challenges in the management of obese patients with colorectal cancer, as well as our proposed recommendations are summarized in Table 10.

In the work-up of patients with colorectal cancer or with colorectal polyps with invasive cancer, modalities such as colonoscopy or ERUS, pelvic MRI, or CT scan may be associated with particular challenges for the obese patient (especially for patients with Class III obesity, i.e., BMI >40). Colonoscopy itself can be difficult in obese patients due to positioning problems, inability to splint effectively, increased scope looping, and higher risk of sedation complications [19]. We recommend that obese patients undergoing colonoscopy should do so in dedicated endoscopy lists with anaesthetic support for sedation or general anaesthesia (GA). The endoscopy room should be set up with a bariatric-size table and have adequate staff available during the procedure to help manoeuvre the patient into different positions, as required. We further recommend that diagnostic colonoscopies (or flexible sigmoidoscopies) in obese patients should be carried out by an interventional gastroenterologist, so that any endoscopic intervention required can take place at the same time, avoiding the need for a second procedure. For obese patients, where endoscopic histological conformation is not possible, we recommend that a CT-PET scan is considered as an alternative.

All cross-sectional imaging modalities (e.g., CT, MRI) have industry standard table weight and aperture limits. In hospitals, the standard table limit of a CT scanner is 205 kg and the limit of an MRI scanner is 159 kg. Specialist bariatric imaging equipment that is currently available includes a weight limit of 308.4 kg for a CT scanner and a weight limit of 249.5 kg for open MRI [20]. We recommend that hospitals should ensure access to centres with bariatric-standard scanners to accommodate the needs of this group of patients. If access to bariatric-standard imaging is limited, then we recommend ERUS as an alternative to CT chest-abdomen-pelvis and pelvic MRI.

It is important to consider the operating room and theatre team set-up for obese patients that are undergoing resectional surgery. We recommend that colorectal surgeons should use a bariatric surgical table or appropriate side table extensions and stirrups for suitable support and comfortable wrapping of the arms at the side. Alternatively, the arm-out position should be used, taking care to avoid over-extension of the shoulders and avoid brachial plexus injuries [21]. The patient should be securely strapped to the table to prevent slipping when in the reverse Lloyd Davis position and abundant gel padding should be used to prevent pressure injuries. The surgeon should consider an optical entry, a technique commonly used in bariatric surgery to achieve intra-abdominal access easily and safely. Bariatric-length trocars and bariatric-length laparoscopic instruments should be available in the operating theatre and should be used if required. Additional 5 mm ports should be considered to allow for more effective assistant retraction and handling of the heavy mesentery [22].

The perioperative pathway should involve pre-operative assessment by an experienced anaesthetist and optimisation in a multidisciplinary setting, with input from surgeons, anaesthetists, dieticians, and physiotherapists. Emphasis should be placed in predicting and assessing individual obese patient risks, e.g., obstructive sleep apnoea, cardiovascular disease, venous thromboembolism (VTE), and diabetes mellitus, which are known to be more prevalent in the obese population. At the time of pre-assessment, in addition to risk assessment, emphasis should be placed in addressing existing comorbidities that may require optimisation e.g., by referral to the Cardiology service for cardiovascular optimisation or referral to the Endocrinology service for optimisation of their diabetic control. Aiming for preoperative weight reduction (POWR) would lead to delays in the surgical pathway and therefore we would not recommend POWR as an appropriate strategy in this group of patients. Preoperative risk prediction with scores such as the Obesity Surgery Mortality Risk Score (OS-MRS), which is already validated in bariatric patients, should be calculated and used to anticipate a patient’s postoperative needs, e.g., critical care unit admission.

The use of a preoperative liver shrinkage diet (low in calories, fat, and carbohydrate) is routinely employed in elective bariatric surgery to reduce liver size and intra-abdominal adiposity. We recommend a preoperative liver shrinkage diet for obese patients planned to undergo MICCP. This would allow for wider intra-abdominal spaces and easier manoeuvrability and therefore improved views and tissue handling for the surgeon. In obese male patients who have a narrow pelvis, use of a robotic minimally invasive approach (by an experienced surgeon) should be considered, as it would allow for better views, access, and pelvic dissection. Obesity is a recognized independent risk factor for SSI [23]. A recent systematic review and meta-analysis (2022) of obese versus non-obese patients undergoing robotic colon surgery noted increased surgical site infections (SSI) in the obese patients but found no significant differences in operative time, conversion to open, or anastomotic leak rates [24]. Stoma formation in this group of patients can be technically challenging due to the thick abdominal wall and/or short mesentery. Patients due to have a defunctioning stoma should undergo preoperative counselling and careful site-marking by the stoma specialist nurse. In obese patients, the stoma site may need to be marked at a higher level than usual to permit adequate visualisation by the patient, as lower stomas may not be visible to the patient when standing. We further recommend that, surgical skill-permitting, an intracorporeal anastomotic technique is preferred (instead of extracorporeal) due to difficulties exteriorising the heavy mesentery, thickness of the abdominal wall and increased risk of incisional hernia in this group of patients [25].

On the day of the surgery, the patient should be anaesthetised by an anaesthetist with experience in bariatric anaesthesia. Anaesthesia should be induced in the head-up ramped position [26]. If possible, the patient should be anaesthetised in-theatre to avoid unnecessary transferring. Alternatively, transfer of the patient should take place on a hover mattress and with the help of additional theatre staff. A ‘difficult airway’ trolley should be present, and the ventilator should have the capability to deliver positive end-expiratory pressure (PEEP) for improved alveolar recruitment. Blood pressure cuff, compression stockings, and Flowtrons of appropriate size should be used.

Post-operatively, due to their pre-existing comorbid profile that puts them at higher risk of complications, we recommend that obese patients would highly benefit from early mobilisation, incentive spirometry or chest physiotherapy, carefully planned and weight-adjusted dosing for venous thromboembolism (VTE) prophylaxis, and antibiotic and analgesia administration.

In the neoadjuvant and adjuvant setting, adjustments to oncological treatments may be required, considering the risks of chemotherapy underdosing (if not carefully adjusted for weight) or of possibly reduced effectiveness of radiotherapy treatment in patients with central obesity. A systematic review (2021) noted that obese patients tolerated full body-size-based dosing of chemotherapy as well as non-obese patients [27]. The American Society of Clinical Oncology (ASCO) guidelines recommend that “full, weight-based cytotoxic chemotherapy doses be used to treat adults with cancer” [27]. A 2018 study noted lower rates of complete response to neoadjuvant chemoradiotherapy, followed by TME in obese patients with rectal cancer. In turn complete response was associated with long-term survival [28]. Therefore, this group of patients may be at higher risk of being undertreated and should be considered for more robust postoperative surveillance. Depending on staging, current guidelines recommend CT chest-abdomen-pelvis every 6 months for 3 years and annually for 2 years in the 5-year surveillance period. We recommend surveillance with CT chest-abdomen-pelvis every 6 months for 5 years, irrespective of stage.

In the context of metastatic disease, obesity does not appear to influence the postoperative mortality and morbidity of cytoreductive surgery and/or HIPEC and therefore should not be contraindicated in obese patients that otherwise meet the criteria for this intervention [29]. Obese patients with obstructing tumours should be considered for palliative resection and diverting stoma where appropriate. However, given the higher complication rates associated with stoma formation in the obese, we recommend that consideration should be given instead to endoscopic stenting, where feasible. Patients with resectable liver and/or lung metastases are managed with upfront resection at the time of the colectomy, or as a second staged procedure. We recommend that obese patients should undergo liver and/or lung resections as a two-staged procedure to reduce prolonged anaesthetic and surgical times, which would pose a physiological burden to the obese patient. Transcutaneous liver ablative techniques may be technically challenging in obese patients due to the thickness of the abdominal wall; these may be more suitable at the time of surgery with direct visualisation and targeting of the liver in the open abdomen.

## 5. Conclusions

In conclusion, the incidence of colorectal cancer and of obesity continues to increase worldwide. Current guidelines do not make provisions for obese patients undergoing MICCP, and modification of the existing guidelines is needed now more than ever to address the complexities of this group of patients. A perioperative multidisciplinary pathway with a focus on risk prediction and risk reduction is of paramount importance to optimise these patients, and the surgical and non-surgical challenges of treating obese patients with colorectal cancer make it highly desirable for a consensus to be reached between colorectal and bariatric surgeons.

## Figures and Tables

**Table 1:** Colon cancer: Precancerous lesions and invasive cancer: assessment and management.

Topic	NCCN ^a^ Recommendations	ESMO ^b^ Recommendations	JSCCR ^c^ Recommendations
**Polyp with Invasive Cancer**			
*Assessment*	Pathology review, colonoscopy and marking of cancerous polyp MMR/MSI testing	Not formally stated	Not formally stated
*Management*	Observe (pedunculated polyp) or colectomy with regional lymphadenectomy (sessile polyp, or incomplete excision)	Observe (pedunculated polyp) Colectomy with regional lymphadenectomy (sessile polyp) or frequent surveillance after endoscopic removal, if surgery not possible due to comorbidities	Polypectomy or snare EMR if <2 cm ESD if 2–5 cm
**Resectable colon cancer**			
*Assessment*	Pathology review, colonoscopy, CEA levels, CT chest-abdomen-pelvis	Pathology review Colonoscopy Blood tests with CEA CT chest-abdomen-pelvis PET-CT not recommended Consider other tests e.g., virtual colonoscopy when complete colonoscopy is not feasible MRI abdomen (to clarify ambiguous lesions or define pT4b)	Not formally stated
*Management*	Colectomy with regional lymphadenectomy +/− diversion or stent if obstructed Consider neoadjuvant chemotherapy or immunotherapy for advanced disease.	Tis/T1N0: local excision >T1N0: colectomy with regional lymphadenectomy pT4b: en block resection of adjacent organ-invaded portions must be carried out Obstructing: one or two-stage procedures Colonic stenting as a bridge to elective surgery in expert centres	Extent of lymphadenectomy (D0–D3) varies with stage (depth of invasion and extent of lymph node metastasis)

Adapted with permission from the NCCN Clinical Practice Guidelines in Oncology (NCCN Guidelines^®^) for Guideline Colon Cancer V.1.2022. © 2022 National Comprehensive Cancer Network, Inc. All rights reserved. The NCCN Guidelines^®^ and illustrations herein may not be reproduced in any form for any purpose without the express written permission of NCCN. To view the most recent and complete version of the NCCN Guidelines, go online to NCCN.org. The NCCN Guidelines are a work in progress that may be refined as often as new significant data become available; EMR = endoscopic mucosal resection; ESD = endoscopic submucosal dissection; CEA = carcinoembryonic antigen; MMR = mismatch repair; MSI = microsatellite instability; 5FU = 5-fluorouracil; RT = radiotherapy; ^a^ NCCN Clinical Practice Guidelines in Oncology (NCCN Guidelines^®^) [13]; ^b^ European Society of Medical Oncology (ESMO) guidelines [15]; ^c^ Japanese Society for Cancer of the Colon and Rectum (JSCCR) guidelines [18].

**Table 2:** Rectal cancer: Precancerous lesions and invasive cancer: assessment and management.

Topic	NCCN ^a^ Recommendations	ESMO ^b^ Recommendations	JSCCR ^c^ Recommendations
**Polyp with Invasive Cancer**			
*Assessment*	Pathology review Colonoscopy Marking of the polyp site MMR/MSI testing	Biopsy Palpation Rigid sigmoidoscopy (flexible endoscopy) Haggitt’s subclassification (if stalked adenoma) Kikuchi (sm) system (if sessile adenoma) ERUS, MRI	Information on size, predicted depth of invasion, and morphology of the tumour
*Management*	Observe (pedunculated polyp) or transanal local excision or transabdominal resection (sessile polyp or if incomplete excision)	Haggitt 1–3, T1 sm1 N0: Local procedure, e.g., transanal endoscopic microsurgery (TEM) Haggitt 4, T1 sm ≥2, high-grade, VI: Radical standard surgery (TME), chemoradiotherapy (if surgery contraindicated) Local radiotherapy as an alternative to local surgery, alone or with (preoperative) chemoradiotherapy	Intramucosal (cTis) or carcinoma with slight submucosal invasion (cT1): Pedunculated: endoscopic polypectomy—up to 2 cm in size Sessile: endoscopic mucosal resection (EMR) or using a cap (EMRC)—up to 2 cm size Endoscopic submucosal dissection (ESD) T1b (depth of Sm invasion ≥1000 μm), lymphovascular invasion positive poorly differentiated, signet-ring cell or mucinous carcinoma, Grade 2/3 budding at the site of deepest invasion: Surgical resection (TME)
**Resectable rectal cancer**			
*Assessment*	Pathology review Colonoscopy CEA levels Chest CT and abdominal CT or MRI Pelvic MRI or ERUS (if MRI is contraindicated, inconclusive, or for superficial lesions) MDT discussion	History Physical exam including DRE Bloods with CEA CT chest-abdomen Rigid sigmoidoscopy Preoperative colonoscopy Virtual colonoscopy in case of obstruction Pelvic MRI ERUS in early cT stage PET-CT if extensive EMVI for other sites MDT discussion	Not formally stated
*Management*	Transanal local excision if appropriate (T1N0) or transabdominal resection (T1-2N0) Total Neoadjuvant Therapy followed by transabdominal resection vs Long-course CRT or SCRT followed by transabdominal resection followed by adjuvant chemotherapy	Very early cT1N0 with low grade G1/G2: → Local excision e.g., TEM → Local RT as an alternative to local excision alone, or combined with CRT Early, not suitable for local excision, T1–2; cT3a (b) if middle or high, N0 (or cN1 if high), -MRF clear, no EMVI: → surgery (TME) alone Intermediate/more locally advanced cT3a/b (very low, levators clear, MRF clear) or cT3a/b (mid or high rectum, cN1-2, no EMVI): → surgery (TME) alone or preoperative RT (CRT or SCPRT) if good quality mesorectal excision cannot be achieved Locally advanced (>cT3b and EMVI+): → surgery (TME) → preoperative RT (CRT or SCPRT)	Tis and cT1: local excision if lesion located distal to the second Houston valve (peritoneal reflection) Extent of lymphadenectomy (D0–D3) varies with stage (depth of invasion and extent of lymph node metastases) TME or tumour-specific mesorectal excision (TSME) Lateral lymph node dissection is indicated when the lower border of the tumour is located distal to the peritoneal reflection and the tumour has invaded beyond the muscularis propria

Adapted with permission from the NCCN Clinical Practice Guidelines in Oncology (NCCN Guidelines^®^) for Guideline Colon Cancer V.1.2022. © 2022 National Comprehensive Cancer Network, Inc. All rights reserved. The NCCN Guidelines^®^ and illustrations herein may not be reproduced in any form for any purpose without the express written permission of NCCN. To view the most recent and complete version of the NCCN Guidelines, go online to NCCN.org. The NCCN Guidelines are a work in progress that may be refined as often as new significant data become available; EMR = endoscopic mucosal resection; ESD = endoscopic submucosal dissection; CEA = carcinoembryonic antigen; MMR = mismatch repair; MSI = microsatellite instability; 5FU = 5-fluorouracil; RT = radiotherapy; ^a^ NCCN Clinical Practice Guidelines in Oncology (NCCN Guidelines^®^) [13]; ^b^ European Society of Medical Oncology (ESMO) guidelines [15]; ^c^ Japanese Society for Cancer of the Colon and Rectum (JSCCR) guidelines [18].

**Table 3:** Colon cancer: Adjuvant treatment.

Pathological Stage	NCCN ^a^ Recommendations	ESMO ^b^ Recommendations	JSCCR ^c^ Recommendations
Stage 0	None	None	None
Stage I	None	None	None
Stage II	Observation or chemotherapy (if high risk features)	Low risk: observation Intermediate risk: 6 months 5FU/leucovorin 6 months Capecitabine High risk: 6 months FOLFOX 3–6 months CAPOX	If high-risk features: Consider chemotherapy after pt counselling
Stage III	Chemotherapy	Low risk: FOLFOX 6 months CAPOX 3 months High risk: FOLFOX 6 months CAPOX 6 months	Options: 5FU, 5FU + leucovorin, UFT, UFT + leucovorin, capecitabine, irinotecan, oxaliplatin, FTD/TPI. Immunotherapy

Adapted with permission from the NCCN Clinical Practice Guidelines in Oncology (NCCN Guidelines^®^) for Guideline Colon Cancer V.1.2022. © 2022 National Comprehensive Cancer Network, Inc. All rights reserved. The NCCN Guidelines^®^ and illustrations herein may not be reproduced in any form for any purpose without the express written permission of NCCN. To view the most recent and complete version of the NCCN Guidelines, go online to NCCN.org. The NCCN Guidelines are a work in progress that may be refined as often as new significant data become available; MMR = mismatch repair; MSI = microsatellite instability; MSI-H = microsatellite instability high levels; dMMR = MMR deficient; pMMR = MMR present; 5FU = 5-fluorouracil; UFT = tegafur and uracil; FTD/TPI = Trifluridine/tipiracil; ^a^ NCCN Clinical Practice Guidelines in Oncology (NCCN Guidelines^®^) [13]; ^b^ European Society of Medical Oncology (ESMO) guidelines [15]; ^c^ Japanese Society for Cancer of the Colon and Rectum (JSCCR) guidelines [18].

**Table 4:** Rectal cancer: Adjuvant treatment.

Topic	NCCN ^a^ Recommendations	ESMO ^b^ Recommendations	JSCCR ^c^ Recommendations
Stage I	None after transabdominal resection	None	None
Stage II & III	Chemotherapy +/− RT Observation is an option for certain T3N0 tumours located in the upper rectum	Postoperative CRT, combined with additional 4 months of adjuvant bolus 5FU. Routine use of CRT has been questioned if a good quality TME can be assured. After surgery alone, consider adjuvant 5FU/leucovorin +/− oxaliplatin	Consider adjuvant chemotherapy in Stage II with high risk of recurrence Adjuvant chemotherapy in Stage III Preoperative RT for patients with cT 3-4 or cN + status Postoperative radiotherapy for patients with pT3-4 or pN + status, where the existence of a surgical dissection plane positive (RM1) or penetration of the surgical dissection plane by the cancer (RMX) is unclear

Adapted with permission from the NCCN Clinical Practice Guidelines in Oncology (NCCN Guidelines^®^) for Guideline Rectal Cancer V.1.2022. © 2022 National Comprehensive Cancer Network, Inc. All rights reserved. The NCCN Guidelines^®^ and illustrations herein may not be reproduced in any form for any purpose without the express written permission of NCCN. To view the most recent and complete version of the NCCN Guidelines, go online to NCCN.org. The NCCN Guidelines are a work in progress that may be refined as often as new significant data become available; 5FU = 5-fluorouracil; RT = radiotherapy; SCRT = short course radiotherapy; CRT = chemoradiotherapy; ^a^ NCCN Clinical Practice Guidelines in Oncology (NCCN Guidelines^®^) [14]; ^b^ European Society of Medical Oncology (ESMO) guidelines [15]; ^c^ Japanese Society for Cancer of the Colon and Rectum (JSCCR) guidelines [18].

**Table 5:** Colon cancer: Postoperative follow-up.

	NCCN^®^^a^ Recommendations	ESMO ^b^ Recommendations	JSCCR ^c^ Recommendations
*History and physical examination*	stage II-IV: every 3–6 months for 2 years, then every 6 months for a total of 5 years	every 3–6 months for 3 years and every 6–12 months at years 4 and 5	every 3 months for 3 years, then every 6 months for 2 years
*Tumour markers*	CEA monitoring, as above	as above	every 6 months for 3 years, then annually for 2 years
*CT chest-abdomen-pelvis*	stage II–IV: every 6–12 months for 5 years (stage IV: every 3–6 months for the first 2 years)	every 6–12 months for 3 years and annually for years 4 and 5	every 6 monthly for 3 years, then annually for 2 years (stage III: every 6 months for 5 years)
*Colonoscopy*	stage I–IV: at 1 year after surgery (except if no preoperative colonoscopy due to obstructing lesion, colonoscopy in 3–6 months). Further colonoscopy intervals determined by findings at 1 year	every 3–5 years starting 1 year after surgery	at 1 year after surgery and at 3 years after surgery

Adapted with permission from the NCCN Clinical Practice Guidelines in Oncology (NCCN Guidelines^®^) for Guideline Colon Cancer V.1.2022. © 2022 National Comprehensive Cancer Network, Inc. All rights reserved. The NCCN Guidelines^®^ and illustrations herein may not be reproduced in any form for any purpose without the express written permission of NCCN. To view the most recent and complete version of the NCCN Guidelines, go online to NCCN.org. The NCCN Guidelines are a work in progress that may be refined as often as new significant data become available; CEA = carcinoembryonic antigen; ^a^ NCCN Clinical Practice Guidelines in Oncology (NCCN Guidelines^®^) [13]; ^b^ European Society of Medical Oncology (ESMO) guidelines [15]; ^c^ Japanese Society for Cancer of the Colon and Rectum (JSCCR) guidelines [18].

**Table 6:** Rectal cancer: Postoperative follow-up.

	NCCN ^a^ Recommendations	ESMO ^b^ Recommendations	JSCCR ^c^ Recommendations
*History and physical examination*	every 3–6 months for 2 years, then every 6 months for a total of 5 years	every 6 months for 2 years	every 3 months for 3 years, then every 6 months for a total of 5 years digital rectal examination every 6 months for 3 years
*Tumour markers*	CEA, as above	every 6 months in the first 3 years	every 6 months for 3 years, then annually for 2 years
*CT chest-abdomen-pelvis*	every 6–12 months for a total of 5 years (stage IV: every 3-6 months for the first 2 years)	minimum of two scans in the first 3 years	every 6 months for 3 years, then annually for a total of 5 years Stage III: every 6 months for 5 years
*Colonoscopy*	at 1 year after surgery (except if no preoperative colonoscopy due to obstructing lesion, colonoscopy in 3–6 months). Further colonoscopy intervals determined by findings at 1 year	completion colonoscopy within the first year if not done pre-operatively colonoscopy with resection of colonic polyps every 5 years up to age 75 years	annually for 3 years
*Additional comments*	Proctoscopy (with EUS), MRI every 3–6 months for a total of 5 years, for patients treated with transanal excision only	In patients who underwent a complete resection of metastatic disease, a more intensive follow-up should be considered: a follow-up with CEA and CT scan at intervals of 3–6 months during the first 3 years can be recommended	In R1 resection, close surveillance schedule should be planned for organs in which residual cancer is suspected

Adapted with permission from the NCCN Clinical Practice Guidelines in Oncology (NCCN Guidelines^®^) for Guideline Rectal Cancer V.1.2022. © 2022 National Comprehensive Cancer Network, Inc. All rights reserved. The NCCN Guidelines^®^ and illustrations herein may not be reproduced in any form for any purpose without the express written permission of NCCN. To view the most recent and complete version of the NCCN Guidelines, go online to NCCN.org. The NCCN Guidelines are a work in progress that may be refined as often as new significant data become available; CEA = carcinoembryonic antigen; ERUS = endoscopic rectal ultrasound; CRC = colorectal cancer; ^a^ NCCN Clinical Practice Guidelines in Oncology (NCCN Guidelines^®^) [14]; ^b^ European Society of Medical Oncology (ESMO) guidelines [16]; ^c^ Japanese Society for Cancer of the Colon and Rectum (JSCCR) guidelines [18].

**Table 7:** Management of metastatic colon cancer.

Site	NCCN ^a^ Recommendations	ESMO ^b^ Recommendations	JSCCR ^c^ Recommendations
Peritoneal	Cytoreductive surgery and/or HIPEC in appropriate cases Systemic therapy +/− resection, diverting ostomy, bypass, or stenting	Cytoreductive surgery and HIPEC	Complete resection for P1 Complete resection for P2 when easily resectable
Liver Lung	*Unresectable:* -systemic therapy	*Resectable liver:* Resection + 6 months adjuvant FOLFOX or perioperative chemotherapy (3 months pre- and 3 months post-resection) *Unresectable liver:* Chemotherapy for downsizing, followed by resection +/− ablative techniques Resect lung metastases if resectable	*Resectable liver:* - synchronous or metachronous resection *Resectable lung:* - metachronous resection

Adapted with permission from the NCCN Clinical Practice Guidelines in Oncology (NCCN Guidelines^®^) for Guideline Colon Cancer V.1.2022. © 2022 National Comprehensive Cancer Network, Inc. All rights reserved. The NCCN Guidelines^®^ and illustrations herein may not be reproduced in any form for any purpose without the express written permission of NCCN. To view the most recent and complete version of the NCCN Guidelines, go online to NCCN.org. The NCCN Guidelines are a work in progress that may be refined as often as new significant data become available; HIPEC = Hyperthermic intraperitoneal chemotherapy; P1 = metastases to the adjacent but not to the distant peritoneum; P2, a few metastases to the distant peritoneum; ^a^ NCCN Clinical Practice Guidelines in Oncology (NCCN Guidelines^®^) [14]; ^b^ European Society of Medical Oncology (ESMO) guidelines [17]; ^c^ Japanese Society for Cancer of the Colon and Rectum (JSCCR) guidelines [18].

**Table 8:** Management of metastatic rectal cancer.

Site	NCCN ^a^ Recommendations	ESMO ^b^ Recommendations	JSCCR ^c^ Recommendations
Peritoneal	Systemic therapy If obstructed or imminent obstruction: Resection or diverting ostomy or bypass or stenting (for upper rectal lesions only)	Complete cytoreductive surgery and HIPEC in appropriate cases. Cytoreductive surgery is particularly effective in patients with low-volume peritoneal disease (PCI *<* 12) and no evidence of systemic disease	*Peritoneal metastases:* - Complete resection is strongly recommended for P1. - Complete resection is recommended for P2 when easily resectable.
Liver Lung	*Resectable:* Neoadjuvant therapy, followed by staged or synchronous resection *Unresectable:* Chemotherapy +/− immunotherapy or targeted therapy +/− SCRT or CRT to convert to resectable	*Resectable liver disease:* -Upfront surgical resection +/− adjuvant FOLFOX (or CAPOX) or -Perioperative FOLFOX *Unresectable liver disease:* - conversion therapy i.e., systemic therapy to convert to resectable disease -local ablative techniques *Lung only:* -ablative techniques if resection is limited by comorbidity, the extent of lung parenchyma resection or other factors *Oligometastatic disease (OMD):* - Treatment strategies based on the possibility of achieving complete removal using surgical resection and/or local ablative treatment (LAT) - For patients with OMD, systemic therapy is the standard of care and should be considered as the initial part of every treatment strategy	*Liver metastases:* -If resectable, liver metastases should be resected upon confirming the radicality of the primary resection. - Simultaneous resection of the primary lesion and liver metastases can be safely performed. - Depending on the difficulty of hepatectomy and the general condition of the patient, metachronous resection is also performed. *Lung metastases:* - If resectable, resection of lung metastases should be considered after resection of the primary tumour. - Metachronous resection is generally performed to remove lung metastases after primary resection.

Adapted with permission from the NCCN Clinical Practice Guidelines in Oncology (NCCN Guidelines^®^) for Guideline Rectal Cancer V.1.2022. © 2022 National Comprehensive Cancer Network, Inc. All rights reserved. The NCCN Guidelines^®^ and illustrations herein may not be reproduced in any form for any purpose without the express written permission of NCCN. To view the most recent and complete version of the NCCN Guidelines, go online to NCCN.org. The NCCN Guidelines are a work in progress that may be refined as often as new significant data become available. 5FU = 5-fluorouracil; RT = radiotherapy; CRT = chemoradiotherapy; SCRT = short course radiotherapy; CRM = circumferential resection margin; PCI = peritoneal carcinomatosis index; HIPEC = Hyperthermic intraperitoneal chemotherapy; P1 = metastases to the adjacent but not to the distant peritoneum; P2, a few metastases to the distant peritoneum; ^a^ NCCN Clinical Practice Guidelines in Oncology (NCCN Guidelines^®^) [14]; ^b^ European Society of Medical Oncology (ESMO) guidelines [17]; ^c^ Japanese Society for Cancer of the Colon and Rectum (JSCCR) guidelines [18].

**Table 9:** Colorectal cancer: additional considerations.

	NCCN ^a^ Recommendations	ESMO ^b^ Recommendations	JSCCR ^c^ Recommendations
Minimally invasive surgery	Considerations: - Experienced surgeon. - No locally advanced disease and/or complications. - Consider preoperative marking of lesion.	Determined by the surgeon’s experience, the stage and location of the cancer and patient factors such as obesity and previous open abdominal surgery	Considerations: - Technical expertise - Location of the tumour - Degree of progression of the cancer - Patient factors: obesity, adhesions

Adapted with permission from the NCCN Clinical Practice Guidelines in Oncology (NCCN Guidelines^®^) for Guideline Colon Cancer V.1.2022. © 2022 National Comprehensive Cancer Network, Inc. All rights reserved. The NCCN Guidelines^®^ and illustrations herein may not be reproduced in any form for any purpose without the express written permission of NCCN. To view the most recent and complete version of the NCCN Guidelines, go online to NCCN.org. The NCCN Guidelines are a work in progress that may be refined as often as new significant data become available; ^a^ NCCN Clinical Practice Guidelines in Oncology (NCCN Guidelines^®^) [13,14]; ^b^ European Society of Medical Oncology (ESMO) guidelines [15,16]; ^c^ Japanese Society for Cancer of the Colon and Rectum (JSCCR) guidelines [18].

**Table 10:** Recommendations for obese patients with colorectal cancer.

	Challenge	Recommendations for Obese Patients
*Diagnostic work up*	Difficult endoscopy Obtaining endoscopic biopsies CT/MRI standard table weight and aperture limits	**For obese patients undergoing endoscopy, we recommend:** (1) Dedicated endoscopy lists, with anaesthetic support and option for GA. (2) A bariatric-size endoscopy table and adequate staffing levels to manoeuvre the patient. (3) The presence of interventional gastroenterologist. **For obese patients, where histological confirmation is not possible, we recommend:** (1) Consider CT-PET as an alternative. **For obese patients undergoing CT or MRI scan, we recommend:** (1) Consider the scanner’s standard table weight and aperture limits. (2) Organise access to centres with bariatric-standard scanners. (3) Consider ERUS as an alternative in obese patients with rectal cancer.
*Anaesthesia*	High-risk airway Associated comorbidities Undiagnosed comorbidities	**For obese patients undergoing anaesthetic pre-assessment, we recommend:** (1) Assessment by an anaesthetist with experience in bariatric anaesthesia and management of difficult airways. (2) Investigation and assessment of known and undiagnosed comorbidies, e.g., diabetes mellitus, cardiovascular disease, VTE, and obstructive sleep apnoea. (3) Appropriate optimisation of comorbidities, e.g., referral to Cardiology for cardiac optimisation. (4) Assess the need for critical care unit admission postoperatively.
*Minimally invasive surgery*	Hepatic steatosis Stoma complications Theatre setup Surgical challenges	**For obese patients undergoing resectional surgery, we recommend:** (1) Preoperative liver shrinkage diet. (2) Preoperative consultation with the stoma nurse specialist if planning to defunction. (3) Preoperative assessment and optimisation by the dietician and physiotherapy team. (4) A bariatric-size theatre table, stirrups and Flowtrons. (5) A hover mattress, (6) Bariatric-size laparoscopic equipment, e.g., bariatric-length ports and long instruments. (7) Consider optical entry. (8) Intracorporeal anastomosis. (9) If available, consider robotic surgery to access the narrow pelvis.
*Postoperative recovery*	High risk of postoperative complications	**For obese patients in the postoperative period, we recommend:** (1) Early mobilization and physiotherapy input. (2) Incentive spirometry +/− chest physiotherapy. (3) Weight-adjusted doses of VTE prophylaxis, antibiotics, and analgesia.
*Adjuvant treatment*	Risk of undertreatment	**For obese patients, undergoing adjuvant treatment, we recommend:** (1) Chemotherapy dosing as per actual body weight, as per the ASCO guidelines.
*Postoperative surveillance*	Need for increased surveillance	**For obese patients, irrespective of staging, and in addition to the surveillance pathways in the current guidelines, we recommend:** (1) Increased frequency of surveillance with CT chest-abdomen-pelvis every 6 months for 5 years.
*Metastatic disease*	Technical and anaesthetic challenges	**For obese patients with metastatic disease, we recommend:** (1) Obesity should not be a contraindication to cytoreductive surgery and/or HIPEC in otherwise appropriate patients. (2) Palliative endoscopic stenting should be considered in obstructing tumours, where feasible. (3) Resection of lung and/or liver metastases should be planned as a two-stage procedure to reduce prolonged anaesthetic and surgical times. (4) Liver ablative techniques may be considered at the time of open abdominal surgery.

## Data Availability

Not applicable.
